# The impact of luteal phase support on endometrial estrogen and progesterone receptor expression: a randomized control trial

**DOI:** 10.1186/1477-7827-10-16

**Published:** 2012-02-24

**Authors:** Paul R Brezina, Nikos F Vlahos, Tsung-Hsuan Lai, Jairo E Garcia, Edward E Wallach, Yulian Zhao

**Affiliations:** 1 Department of Gynecology and Obstetrics, Division of Reproductive Endocrinology and Infertility, Johns Hopkins University School of Medicine, 600 N. Wolfe Street, Baltimore, USA; 2 Department of Obstetrics and Gynecology, Division of Reproductive Endocrinology and Infertility, Athens University School of Medicine, Athens, Greece; 3 Department of Obstetrics and Gynecology, Fu Jen Catholic University School of Medicine, HsinChu Cathay General Hospital, New Taipei and HsinChu Cities, Taiwan

**Keywords:** ERα, ER alpha, Endometrium, "Controlled ovarian hyperstimulation"

## Abstract

**Background:**

To assess the impact of luteal phase support on the expression of estrogen receptor (ER) alpha and progesterone receptors B (PR-B) on the endometrium of oocyte donors undergoing controlled ovarian hyperstimulation (COH).

**Methods:**

A prospective, randomized study was conducted in women undergoing controlled ovarian hyperstimulation for oocyte donation. Participants were randomized to receive no luteal support, vaginal progesterone alone, or vaginal progesterone plus orally administered 17 Beta estradiol. Endometrial biopsies were obtained at 4 time points in the luteal phase and evaluated by tissue microarray for expression of ER alpha and PR-B.

**Results:**

One-hundred and eight endometrial tissue samples were obtained from 12 patients. No differences were found in expression of ER alpha and PR-B among all the specimens with the exception of one sample value.

**Conclusions:**

The administration of progesterone during the luteal phase of COH for oocyte donor cycles, either with or without estrogen, does not significantly affect the endometrial expression of ER alpha and PR.

## Background

The spatial and temporal expression of specific extracellular matrix (ECM) proteins and adhesion molecule genes creates a profile that is crucial for successful embryo implantation [1,2]. The effect of exogenous hormone administration on these complex hormonal signaling pathways is not totally elucidated. Some investigators purport that controlled ovarian hyperstimulation (COH) protocols inevitably lead to lack of synchrony between the development and maturation of the endometrium and the time of oocyte retrieval or "ovulation" [3,4]. However, conflicting information exists as to whether COH does in fact lead to a clinically significant degree of endometrial lack of synchrony [5-8].

Ovarian stimulation protocols for IVF have been frequently associated with luteal phase deficiency and poor implantation rates [3,4]. Additionally, there are some data showing that, in GnRH agonist/HMG stimulated cycles, lack of supplementation with exogenous progesterone (P) results in impaired P bioavailability [9,10]. For this reason, luteal phase support is customarily used to improve endometrial structure and histology thus facilitating the implantation process. P is accepted as the preferred agent for luteal phase support and is administered orally, intramuscularly, or vaginally [11,12].

Animal and human studies have shown that estradiol (E2) serves a critical role in endometrial development compatible with successful pregnancy [13,14]. Additionally, data have shown that, in some women, E2 levels fall in the mid luteal phase of an IVF cycle [14]. This finding led to the incorporation of 17b-E2 or E2 valerate into many ART programs for luteal phase support [3,4].

Studies evaluating the concept of E2 addition during the luteal phase have thus far failed to show any benefit or led to inconclusive results [3,4,10,15]. However, data regarding the effect of luteal phase support in IVF cycles are limited and there is still not a universal consensus regarding optimal supplementation during the luteal phase [12,16,17].

In the present study, we investigated the effects of two commonly used luteal phase support protocols (P alone and P plus E2), on the expression of the E2 Receptor α (ERα) and the P receptor B (PR-B) in the human endometrium following ovarian stimulation with a gonadotropin/GnRH antagonist protocol.

## Methods

### Clinical trial

The study was approved by the Johns Hopkins Hospital Institutional Review Board.

Women undergoing COH for oocyte donation in an outpatient assisted reproductive technology (ART) clinic were enrolled over the period of 1 year. A power analysis was not performed prior to initiating the study. Rather, all eligible women possible were approached regarding study participation. Women from 21-29 yr of age were eligible as oocyte donors. The selection process included a thorough questionnaire and psychological evaluation of the potential donors followed by a detailed physical examination and consultation to discuss the process of oocyte donation by a physician member of the group. The risks of the procedure were discussed in detail, and written informed consents were obtained. All donors were screened for sexually transmitted diseases as well as for genetic conditions in accordance with the recommendations of the American Society for Reproductive Medicine [18]. Women with a body mass index exceeding 28 kg/m^2^, history of pelvic inflammatory disease, sexually transmitted diseases, reproductive tract pathology, or other systemic diseases or conditions were excluded. No women included in this study had any known endocronlogic abnormality including polycystic ovarian syndrome. At the time of their initial visit, they received a detailed explanation of the study protocol with particular emphasis on the risks associated with the endometrial biopsy and the use of steroids during their luteal phase. A written informed consent was obtained at that time.

Oocyte donors were stimulated with a GnRH antagonist protocol. Briefly, all donors had a baseline measurement of FSH and E2 serum concentrations on the second day of their menstrual cycles after the discontinuation of oral contraceptive pills. In addition, a transvaginal sonogram was performed to rule out early follicular development and any anatomic anomalies. Providing that serum FSH was less than 10 mIU/ml and E2 was less than 60 pg/ml, ovarian stimulation was initiated with 225 IU recombinant FSH (Follitropin Alfa, Gonal-F; Serono Laboratories, Norwell, MA). A daily evening dose of ganirelix acetate (Antagon; Organon, West Orange, CA), 0.25 mg sc, was started either 6 d after the initiation of gonadotropins or at the time of identification of a leading follicle with mean diameter more than 13 mm and continued through the day of human chorionic gonadotropin (hCG). Thereafter, the dose of gonadotropins was adjusted in a step-down fashion according to follicular development by serial transvaginal ultrasound and serum E2 response. When at least three follicles reached a mean diameter of 18 mm, ovulation was triggered with a single im dose of 10,000 IU hCG (Profasi; Serono) or 20 U of a GnRH agonist administered in two doses 24 h apart. Transvaginal oocyte retrieval was performed under iv sedation 34-36 h after hCG or the initial dose of GnRH agonist.

**Figure 1:**
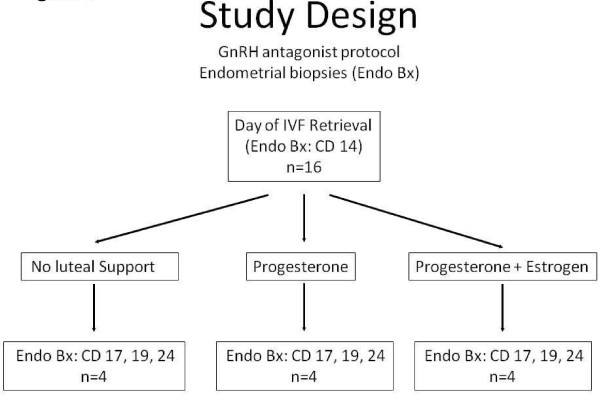
**Study design**. This figure outlines the design of the study. The baseline endometrial biopsy is shown at the top of the graph. The 3 arms of the study are then shown and described in each of 3 boxes.

On the day of oocyte retrieval, the study participants were randomized into three treatment categories: The "none" group did not receive any luteal phase support; the "P" group received micronized P in the form of vaginal suppositories (200 mg every 6 h starting from the day after retrieval); the "P + E" group received a daily oral dose of 2 mg 17b-E2 in addition to the micronized P. (Figure 1) Up to that point, all donors had been stimulated according to the same protocol and had received a comparable amount of medication. An endometrial biopsy on the day of oocyte retrieval was performed on all study participants to define a baseline status for the endometrium.

Endometrial biopsies were performed using a Pipelle catheter (Unimar, Wilton, CT) on the day of oocyte retrieval (cycle day [CD] 14 of the benchmark cycle) and then 3, 5, and 10 days after oocyte retrieval corresponding to ideal cycle days 17, 19, and 24. At least 2-4 endometrial biopsies were obtained from each donor. The tissue in the endometrial samples were identified as either luminal epithelium, the surface epithelium lining the uterine cavity, glandular epithelium, or stromal tissue. The specimens were then fixed in 10% formalin, and subsequently embedded into paraffin for tissue microarray sectioning.

### Microarray analysis

**Figure 2:**
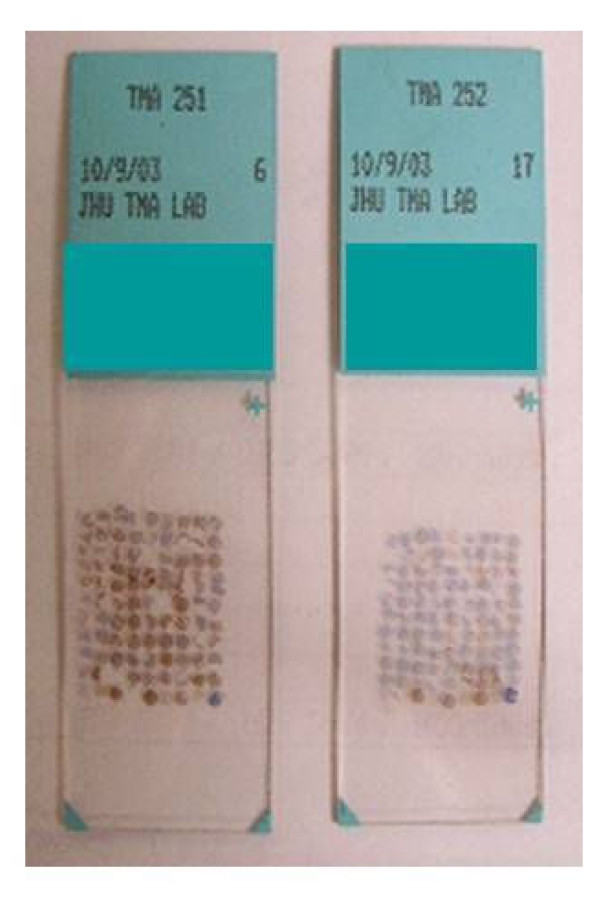
**Representative microarray slide**. This picture shows the tissue cores from the samples affixed to the tissue microarray slides used in this study.

Tissue microarrays (TMA) were assembled from paraffin embedded endometrial tissues in a 1.5-mm diameter for each tissue sample. Three representative tissue samples were obtained for each specimen. The arrays encompassed 108 tissue cores derived from 12 donors (Table 1). All tissue cores were sectioned in 5-μm thickness and affixed to the TMA slides. (Figure 2) The expression of ERα and PR-B were examined using Confirm antiER (6F11) and Confirm anti-PR [16] mouse monoclonal antibodies (Ventana Medical Systems Inc, Tucson, Arizona) directed against the human ER and PR molecules. The tissue sections were then incubated with biotinylated goat antimouse immunoglobulin. Positive immunostaining was detected through interaction of avidin-biotin perioxidase (ABC) complex with biotin-conjugated secondary antibody using a Ventana DAB Detection Kit (Ventana- Biotek Solutions, Tucson, AZ). Isotype-specific irrelevant monoclonal antibody MiTF, generated against the human microthalmia transcription factor in melanoma, was used as a primary antibody for a negative control [19]. Slides were subsequently counterstained with hematoxylin. The intensity of immunostaining was assessed using the HSCORE as described previously [20,21]. Statistical analysis was performed using the Mann-Whitney U test and Student *t *test when appropriate using statistical package SPSS (Chicago, IL). Significance was determined by a *P *value of ≤ 0.05.

**Table 1:** Endometrial progesterone receptor B expression in different luteal phase support groups

		Group None	Group P	Group P+E	P Value
		H score (mean score)			
CD 14	Luminal	7.6 (n = 4)	5.8 (n = 4)	7.9 (n = 4)	0.60
	Glandular	9.5 (n = 4)	7.0 (n = 4)	6.4 (n = 4)	0.49
	Stroma	6.9 (n = 4)	6.3 (n = 4)	8.0 (n = 4)	0.75
CD17	**Luminal**	**3.8 **(n = 3)	**9.3 **(n = 3)	**4.4 **(n = 3)	**0.04**
	Glandular	6.7 (n = 3)	8.3 (n = 3)	3.3 (n = 3)	0.09
	Stroma	6.8 (n = 3)	7.4 (n = 3)	5.1 (n = 3)	0.63
CD19	Luminal	4.8 (n = 3)	5.0 (n = 2)	5.1 (n = 3)	0.99
	Glandular	4.5 (n = 3)	6.8 (n = 2)	5.6 (n = 3)	0.71
	Stroma	5.8 (n = 3)	6.3 (n = 2)	5.0 (n = 3)	0.85
CD24	Luminal	3.0 (n = 2)	4.7 (n = 2)	7.0 (n = 3)	0.1 9
	Glandular	3.5 (n = 2)	8.0 (n = 2)	4.8 (n = 3)	0.13
	Stroma	5.8 (n = 2)	4.5 (n = 2)	6.0 (n = 3)	0.78

This table shows the H score for the Progesterone Receptors evaluated in this study. Statistical differences, at a level of ≤ 0.05, between the H score values for the various groups (natural, Estrogen supported, and Estrogen and Progesterone supported) are also provided and H score values that significantly differ between groups are bolded.

## Results and discussion

Endometrial biopsies were obtained from 12 oocyte donors, randomized into 3 groups of 4 patients each. There were no differences in the age (average of 24.1 ± 1.0) or BMI (average of 21.1 ± 0.6 kg/m2). There were no significant differences among the donors regarding serum hormone profiles. No difference was found in baseline laboratory values, including follicle stimulating hormone (FSH) and E2 levels on CD#2, or the total injectable medications (number of FSH ampoules and dose of Ganirelix Acetate) used among the groups. Peak serum E2 levels ranged from 1,817 pg/ml to 2,547 pg/ml with no differences among the 3 groups (p = 0.378). Immediately prior to oocyte retrieval, transvaginal ultrasound measurement of the endometrial lining of all patients ranged between 9 and 11 mm and did not differ among the groups. No difference was noted in the number of oocytes retrieved (range of 14 to 19) among the study groups (p = 0.367).

No difference was found in the expression of the ERα and PR between the "None", "P", and "P + E" groups in all but one time point in luminal endometrium,. There were no differences in ERα values noted among all groups evaluated at all biopsy time points. The only significant difference in PR-B expression was observed on CD 17 in the luminal epithelium of endometrium (Table 1). In this case, the "P" group showed increased PR-B expression as compared to the "none" or "P + E" groups at a level of p = 0.04 in the luminal endometrium. However, even in this group, there was no difference noted in PR-B expression in the glandular and stromal endometrium between the groups. This single difference is likely due to chance as the study sample size was small. The difference could not be explained by other clinical parameters, as all women were without medical illness or other pathology prior to and during the study.

E2 is well accepted to be a critical component of endometrial development and pregnancy in both animal and human models [13,15]. Some studies have shown that, in some women, E2 levels may drop during the luteal phase of an IVF cycle [14]. However, the supplementation to P for luteal support with exogenous E2 in IVF cycles has generally failed to affect significantly ultimate pregnancy rates [3,4]. Our data supports these clinical findings, as we did not find an effect on ERα and PR-B expression when exogenous E2 was added to luteal phase support.

This study evaluated the endometrial expression of PR-B. There are 2 distinct isoforms of PR (A and B) [22,23]. PR-B, which has an additional 164 amino acids at its amino terminus, expression results in a stimulatory effect on P target genes [23]. ERα and PR-B have stimulator effects on stromal, glandular, and luminal endometrium [24]. In the natural cycle, PR-B expression increases in the luminal epithelium and in the stroma during the proliferative phase and then remains high well into the secretory phase [24]. However, in the glandular epithelium many studies have shown a decline in PR-B expression during the late secretory phase immediately prior to menses [24]. Furthermore, in the natural cycle, P has been shown to have a down-regulatory effect on PR-B. This study was primarily interested in the effect of possible downregulation of the PR-B receptor in response to exogenous P and E2 exposure. In the IVF setting, and with luteal support, we did not observe a down regulation of PR-B expression in the late luteal phase. PR-A actually suppresses PR-B expression. In the future, similar studies should consider evaluating the expression of PR-A. Specifically, PR-A expression in glandular epithelium has been documented to be associated with endometrial apoptosis [25]. Evaluating PR-A, in addition to PR-B, in future studies may enhance the findings of this and other studies evaluating the effect of exogenous horomones on endometrial function.

A chief limitation of this study is the low number of oocyte donors included in the analysis. This is likely a consequence of the difficulty in recruiting women agreeable to participate in a study requiring inconvenient follow up appointments and an invasive procedure (endometrial biopsy). The low number of participants in this study could have led to spurious results. Indeed, it is possible that larger sample sizes could have identified differences in these various populations. However, we could not detect trends in the data that would suggest that such differences would have been found with increased sampling size. Certainly future studies with larger numbers of participants would help to further assess the results of this study.

Endometrial receptivity is vital to the process of implantation. Thus investigators have begun to study in detail the complex signaling cascades necessary for endometrial development and their responses to exogenous hormonal manipulation [26]. Luteal phase hormonal support is almost universally used in IVF cycles to enhance implantation of transferred embryos [27]. In this report, we have demonstrated that the administration of vaginal P, with and without E2, does not appear to significantly affect PR expression in the endometrium in all but one time point in luminal epithelium. We could not identify any plausible explanation, based on either background research or demographic characteristics of the patients in this trial, to reconcile the presence of this outlier result.

## Conclusions

Our observation is reassuring, supporting the use of luteal phase support with P, as this practice has no deleterious effect on the expression of PR in the uterine endometrium. Indeed, it would not be unreasonable to expect that exogenous P administration could lead to a downregulation of uterine PR receptors. Whether the addition of E2 to the regimen has any additional effect needs further investigation. Further studies will undoubtedly delineate the endometrial changes induced through COH cycles. The results of this study are limited in evaluating the possible adverse effects of the hormone therapies described. As the women evaluated in this study were all oocyte donors and did not undergo uterine transfer of an embryo, the effect of these medications on a pregnancy, based on our data alone, is not possible to quantify. Furthermore, an intrauterine pregnancy would likely change the endometrial and hormonal environment and thus compromise the application of our results in pregnant patients. However, our results are reassuring given that, in the oocyte donor population, down regulation of ERα and PRB was not observed with exogenous hormone luteal support. Understanding the relationships between COH/luteal support and endometrial maturation can lead to further refinement and optimization of IVF protocols.

## Competing interests

The authors declare that they have no competing interests.

## Authors' contributions

NV, JG, EW were directly involved in patient care and obtaining samples used in the trial. PB, EW, NV, TL, YZ were all involved in interpreting data and involved in composition of the paper. All authors read and approved the manuscript.
